# Hyperthyroidism Masquerading as an Anxiety Disorder: A Report on a Misdiagnosed Case

**DOI:** 10.7759/cureus.44071

**Published:** 2023-08-24

**Authors:** Serin Moideen Sheriff, Gauravdeep Singh, Nonso Kingsley Chukwunyelu, Chinedu J Ezeafulukwe, Omar A Hassan

**Affiliations:** 1 Department of Emergency Medicine, Lal Bahadur Shastri Hospital, New Delhi, IND; 2 Department of Internal Medicine, Odesa National Medical University, Odessa, UKR; 3 Department of Internal Medicine, Kasturba Medical College, Manipal, IND; 4 Department of Internal Medicine, Sumy State University, Sumy, UKR; 5 Department of Internal Medicine, Windsor University School of Medicine, Cayon, KNA; 6 Department of Internal Medicine, Ondokuz Mayis University, Samsun, TUR

**Keywords:** generalized anxiety disorder, anxiety disorder, thyrotoxicosis, grave’s disease, hyperthyroidism

## Abstract

Hyperthyroidism is an endocrine disorder characterized by excess thyroid hormone production. Its classic symptoms include weight loss, palpitations, tremors, and anxiety. We present a case of a 33-year-old female who initially presented with anxiety-like symptoms, leading to a misdiagnosis of generalized anxiety disorder. Upon further evaluation, the patient was diagnosed with hyperthyroidism, highlighting the importance of evaluating endocrine causes in patients presenting with anxiety-like symptoms. This case report underscores the significance of a comprehensive medical evaluation in patients with anxiety to avoid misdiagnoses and ensure appropriate management.

## Introduction

Hyperthyroidism is a medical condition marked by an excessive presence of thyroid hormones in the bloodstream due to an overactive thyroid gland with heightened hormone production ​[1]​. This condition can manifest due to various factors, including Graves' disease (GD), toxic multinodular goiter, thyroiditis, toxic solitary nodules, and even medication effects. Among these, GD emerges as the primary contributor to this condition ​[1]​. Diagnosis often involves observing heightened levels of thyroxine (T4) and triiodothyronine (T3) in the serum, coupled with diminished levels of thyroid-stimulating hormone (TSH). The term *thyrotoxicosis* is employed when there is an excessive thyroid hormone presence in the circulation, regardless of its underlying cause ​[1]​.

Anxiety disorders are characterized by persistent and potentially escalating anxiety, which can significantly impact daily functionality. Such symptoms may disrupt tasks like work, schooling, and social interactions, prompting many to seek medical intervention to address the repercussions of anxiety ​[2]​. Restlessness, trembling, sweating, rapid breathing, increased heart rate, fatigue, difficulty concentrating, disrupted sleep, gastrointestinal issues, uncontrollable worry, and an inclination to avoid anxiety-inducing stimuli are prevalent signs of anxiety disorders ​[2,3]​. Interestingly, these manifestations bear a resemblance to hyperthyroidism symptoms. In certain instances, these symptoms might obfuscate underlying medical conditions, thereby introducing complexities in the diagnostic process [3]​. The present case report delineates a distinct scenario wherein hyperthyroidism masqueraded as an anxiety disorder. This underscores the significance of considering endocrine origins in patients who present with anxiety-like symptoms.

## Case presentation

A 33-year-old female patient visited the outpatient psychiatry clinic with persistent complaints of restlessness, difficulty sleeping, fatigue, and palpitations over the past six months. She conveyed a constant feeling of being *on edge* and recounted frequent episodes of panic attacks. Notably, the patient had no significant past medical conditions, and she denied experiencing fever, chest pain, shortness of breath, or weight loss. There was a significant psychiatric, endocrine, or autoimmune history in her family. She was a nonsmoker and abstained from alcohol. During the physical examination, the patient exhibited signs of anxiety and worry. Her mucous membranes were adequately moist, and her abdomen was soft and nontender upon palpation. Cardiac auscultation revealed normal heart sounds without any murmurs, while the bilateral lung fields exhibited clarity. Vital signs measured her blood pressure at 130/78 mmHg, heart rate at 92 beats per minute, respiratory rate at 16 breaths per minute, temperature at 98 °F, body mass index (BMI) at 20.2 kg/m², and oxygen saturation in room air at 99%. Importantly, she displayed no hand tremors, and exophthalmos was not present during the examination. A psychiatric assessment was conducted using a hospital-based Generalized Anxiety Disorder-7 (GAD-7) screening tool as depicted in Table 1. The total score was 11, which led to the diagnosis of moderate GAD. Consequently, a daily prescription of oral paroxetine (selective serotonin reuptake inhibitors, or SSRIs) at a 20 mg dose was started, alongside the initiation of cognitive behavioral therapy (CBT) was advised as a part of her treatment plan.

**Table 1:** Generalized Anxiety Disorder-7 screening chart. Total score 11 (moderate anxiety disorder). Cutoff scores 5, 10, and 15 represent mild, moderate, and severe anxiety disorder, respectively

Over the past two weeks, how often the patient has been feeling bothered by the symptoms	Not at all (0 points)	Several days (1 point)	More than half the days (2 points)	Everyday (3 points)
Feeling nervous, anxious, or on edge				3
Not being able to stop worrying		1		
Worrying too much about different things		1		
Trouble relaxing			2	
Restless, that it is hard to sit still			2	
Easily annoyed or irritable			2	
Feeling afraid as if something awful might happen	0			

One month later, the patient returned for a follow-up visit. When discussing the improvement of symptoms, she reported that there was no alleviation and that her distress persisted. Upon further questioning, she disclosed unintentional weight loss of approximately 8 pounds in the past month, coupled with an increased appetite. She denied any heat intolerance, alterations in the menstrual cycle, or changes in bowel habits. During the physical examination, fine tremors were observed in both hands, accompanied by sweating. No neck swelling was evident, and vital signs remained normal except for a heart rate of 102 beats per minute. Considering the atypical features of the patient's anxiety-like symptoms, and her recent weight loss led to a comprehensive medical evaluation to be done. Laboratory tests were carried out, encompassing a complete blood count (CBC), comprehensive metabolic panel (CMP), urine pregnancy test, and thyroid function tests. All results fell within normal ranges except for thyroid function tests, which exhibited low TSH levels high free T3 levels, and high free T4 levels, thereby confirming the diagnosis of hyperthyroidism. The results of the Thyroid Function Test are presented in Table 2. Aiming to ascertain the etiology of the hyperthyroid state, blood tests were conducted to determine serum thyroid antibody levels. The results indicated elevated titers of TSH receptor antibody and thyroid peroxidase antibody, conclusively diagnosing GD. Table 3 shows the results of the thyroid antibody test.

**Table 2:** Thyroid function test. TSH, thyroid stimulating hormone; T3, triiodothyronine; T4, thyroxine

Component	Patient value	Normal range
Free T3 (pmol/L)	22	3.52-6.62
Free T4 (pmol/L)	34	11.58-21.88
TSH ( mIU/mL)	<0.06	0.50-5.0

**Table 3:** Thyroid antibody test. TRAb, thyroid-stimulating hormone receptor antibody; TPO, thyroid peroxidase antibody

Component	Patient value	Reference range
TRAb (IU/L)	8.6	<1.5
TPO Ab ( IU/mL)	74	<9

The patient was subsequently referred to an endocrinologist for further evaluation and management. The treatment strategy encompassed a daily oral methimazole tablet at a 30 mg dosage (an antithyroid medication) to regulate thyroid hormone production. Additionally, to alleviate tremors and tachycardia, an oral propranolol tablet (a beta-blocker) at 40 mg daily was also prescribed. Responding positively to the treatment, the patient's anxiety-like symptoms gradually ameliorated, while her weight increased as her thyroid hormone levels normalized. Regular monitoring, including monthly thyroid function tests, was instituted, allowing for a gradual tapering of the methimazole dosage to 5 mg daily, thereby maintaining optimal thyroid hormone levels.

## Discussion

When delving into the connection between thyroid disorders and mental health conditions, it becomes essential to take into account thyroid hormone metabolism within the brain. The hypothalamic-pituitary-thyroid (HPT) axis encompasses intricate interactions involving multiple elements such as thyroid hormones, deionization enzymes, transport proteins, and receptors [​4]. Gaining a detailed comprehension of each of these components proves valuable in elucidating the origins of mental illnesses and optimizing their treatment [4,5].​

The monoamine hypothesis, which underscores the relationship between mental disorders and the activity of monoamine neurotransmitters from a biological standpoint, emerges as a strong contender. Pertinently, in terms of thyroid hormone interplay, T3 is known to regulate serotonin and noradrenaline levels [4,5]. In a study conducted by psychiatrist Fujinami [6], involving 325 patients, a significant association between thyroid conditions and psychiatric disorders was highlighted. Specifically, he reported a prevalence of 63.1% for GD and 10.5% for Hashimoto's disease (HD) among patients with psychiatric concerns ​[6]. Another study by Iacovides et al. examined 50 patients diagnosed with both GAD and hyperthyroidism. This study concluded that a careful analysis of clinical symptomatology could potentially distinguish the two conditions [7]. Furthermore, Winsa conducted a case-control study that showed an association between stress and GD. In comparison to the control group, GD patients reported a greater frequency of stressful life events, with significantly elevated scores ​[8]. All of these observations suggest a potential link between thyroid function imbalances and mental irregularities. A meta-analysis demonstrated that the impact of T3 enhances these neurotransmitters ​[9]. Other studies reported that a decrease in serotonin and noradrenaline is correlated with depression and anxiety, and reduced T3 levels can lead to depressive and anxiety disorders ​[10,11].

Several other potential factors contributing to misdiagnosis, in this case, could be linked to the association of GAD with various elements, including female sex, unmarried status, lower level of education, the median age of onset at 30 years, poor health, and significant life stressors [12]. Even though there are studies that report the correlation between thyroid disorders and psychiatric conditions, consistent and timely diagnostic approaches are yet to be introduced in the clinical setting. Instances of misdiagnosis persist, pointing toward the importance of raising awareness. This case report highlights the significance of considering endocrine disorders, particularly hyperthyroidism, in patients who present with anxiety-like symptoms.

## Conclusions

Hyperthyroidism can manifest with anxiety-like symptoms, mimicking psychiatric disorders such as GAD. The overlapping symptomatology between these conditions can foster misdiagnosis and improper management of the underlying medical ailment. Recognizing and assessing thyroid function on time is pivotal, particularly in instances of atypical or treatment-resistant anxiety disorder. Apart from psychological evaluation, comprehensive medical evaluation, including a physical examination and appropriate laboratory tests, are to be performed in patients who present with anxiety-like symptoms.
